# Anti-N-methyl D-aspartate Receptor Encephalitis Following ChAdOx1 nCoV-19 Vaccination: A Case Report

**DOI:** 10.34172/aim.2023.87

**Published:** 2023-10-01

**Authors:** Zahra Liyaghatdar, Amirhosein Rahimkhani, Amin Liaghatdar

**Affiliations:** ^1^Department of Biochemistry, Faculty of Biological Sciences, Tarbiat Modares University, Tehran, Iran; ^2^Faculty of Medical Sciences, Zahedan University of Medical Sciences, Zahedan, Iran

**Keywords:** Anti-NMDAR encephalitis, Autoimmune encephalitis, Case report, ChAdOx1 nCoV-19 vaccine

## Abstract

As of December 2020, millions of people have been immunized using vaccines against SARS-CoV-2. A wide range of neurological adverse effects of SARS-CoV-2 vaccines have been reported so far. Here, we report a 23-year-old male who experienced psychiatric symptoms, loss of consciousness, language disintegration, and incontinency that happened 10 days after the first dosage of the COVID-19 AstraZeneca vaccine. Anti-NMDAR Encephalitis was diagnosed based on the results of the autoimmune panel. The patient responded to intravenous dexamethasone very well and experienced no other complications in 6 months of follow-up. Scientific reports of neurological side effects such as anti-NMDAR encephalitis after vaccination are necessary to optimize the safety and effectiveness of SARS-CoV-2 vaccines.

## Introduction

 Autoimmune encephalitis refers to a relatively common cause of neurological inflammation disease correlated with autoantibodies. An increasing number of autoantibodies associated with encephalitis have been reported, each with its own clinical manifestation. Anti-N-methyl D-aspartate receptor (Anti-NMDAR) encephalitis is currently the most common cause of autoimmune encephalitis.^1,2^

 Anti-NMDAR encephalitis is usually described as a syndrome of psychiatric manifestations, language disintegration, seizures, memory deficits, orofacial dyskinesia, movement disorder, coma, and autonomic and breathing instability. This autoimmune encephalitis can be associated with tumors and the commonly associated tumor is ovarian teratoma. Methylprednisolone and intravenous immunoglobulin (IVIG) or plasma exchange, and tumor removal if present, are the first line of treatment for this autoimmune disorder. Steroids, rituximab, cyclophosphamide, and other immunosuppressive drugs could also be prescribed.^3^

 Millions of people worldwide were affected by the COVID-19 pandemic. Due to this fact, several vaccines have been designed and developed and have received emergency approval from Food and Drug Administration (FDA).^4^ The Oxford-AstraZeneca or AZD1222 COVID-19 vaccine, an adenovirus-based vaccine, was first approved in late December 2020 in the United Kingdom and then in many other countries.^5^

 Neurological complications following ChAdOx1 nCoV-19 vaccination have been reported many times just like other COVID-19 non-viral vaccines and many non-COVID-19 viral vaccines that have been well-reviewed by Aliasin and colleagues.^6^ Nevertheless, we have not seen any case report with anti-NMDAR encephalitis after ChAdOx1 nCoV-19 vaccination so far.

 In this document, we report a 23-year-old man with anti-NMDAR encephalitis following the first dose of ChAdOx1 nCoV-19 vaccination.

## Case Report

 A 23-year-old medical student was brought to the emergency unit by his friends with agitation and disorientation without any medical problems before. His primary symptoms were mild bitemporal headache and drowsiness that was followed by a severe retro-orbital headache, delusion, language disintegration, automatism, charge and discharge consciousness, and incontinency. He told his friends that this is the worst headache he had ever experienced and felt something heavy moving in his head with any motion of the head. No seizures or breathing instability were mentioned. The patient’s friends mentioned that they were all vaccinated against COVID-19 with the AstraZeneca ChAdOx1 nCoV-19 vaccine 10 days ago and it was their first dosage.

 We saw the evidence of psychosis in the first view. A mildly decreased blood pressure (105/75) was detected on admission and other vital signs were normal (PR = 85, RR = 15, T = 37.3). He was agitated and had to be controlled with haloperidol. We also found some purple striae on his upper arm that seems to be from past and also gynecomastia. He was hospitalized with the initial diagnosis of acute delirium and suspicion of drug abuse.

 Drug tests such as opium, cocaine (COC), amphetamine (AMP), methamphetamine (MET), tetrahydrocannabinol (THC), methadone (MTD), morphine (MOP), phencyclidine (PCP), barbiturates (BAR), benzodiazepines (BZO), and tricyclic antidepressants (TCA) were all negative. Laboratory tests showed 3 + positive C-reactive protein (CRP), but complete blood count (CBC), diff, Trop (troponin), and biochemistry tests were all normal. Blood culture, COVID-19-PCR, and other blood tests were negative. ECG was normal. Brain CT scan, and brain MRI were performed on a suspicion of viral encephalitis and meningitis. CT scan and MRI were normal; lumbar puncture (LP) only revealed mildly elevated cerebrospinal fluid (CSF) pressure and was negative for COVID-19, HSV1, HSV2, HIV, TB, and other infections.

 He had no progress towards remission by receiving acyclovir, ceftriaxone, vancomycin, and Dilantin. Chest CT, pelvis and abdomen sonography reported nothing abnormal, except some renal microlithiasis, but EEG reported generalized slowing and sharp spike waves that suggested non-convulsive epilepsy. Positron emission tomography scan also reported nothing abnormal. The bone mineral density of anteroposterior spine and femur was measured and reported mild osteopenia.

 After that, 8 mg dexamethasone every 8 hours was initiated for the patient on a suspicion of autoimmune encephalitis and serum autoimmune panel was ordered. Another LP was also ordered but could not be performed because of the area’s instability. After two days, the patient’s consciousness improved very well and the glutamate receptors test (type NMDA) reported positive with 1/10 titration although LGL1, CASPR2, DPPX, and the rest of the autoimmune panel came back negative. Another control test for NMDAR was ordered one week later and reported positive again with the same titration (the patient did not consent to the second LP).

 Finally, he received 500 mg methylprednisolone twice a day IV for 3 days with diagnosis of anti-NMDAR encephalitis, and discharged from the hospital with oral prednisolone 50 daily, which was tapered over 4 months, alendronate 70 and Calcium D because of mild lumbar osteopenia and receiving corticosteroid, Phenytoin 100 TDS which became BID after the second EEG was normal, and continued for 2 months, and then turned daily for 6 months. The patient reported no more problems except many papular rashes on his back that were confirmed as a complication of corticosteroids and did not last long.

## Discussion

 Here, we report for the first time an anti-NMDAR encephalitis in a 23-year-old man whose symptoms occurred 10 days following the first dosage of the Covid-19 AstraZeneca.

 Based on the Grauscriteria, possible autoimmune encephalitis was defined by acute onset of psychiatric symptoms that were related to vaccination, CSF pleocytosis, seizures without a prior history, exclusion of other causes, and response to immunosuppressive drugs.^1,7^ It has been reported frequently that brain MRI is unremarkable in 50% of patients with anti-NMDAR encephalitis; in contrast, EEGs are abnormal in most patients usually showing non-specific, slow, and disorganized activity, sometimes with electrographic seizures, as we saw in this case. The most reliable diagnostic test is examination of the CSF following LP to assess anti-NMDAR antibodies in the sample. In most cases, it shows moderate lymphocytic pleocytosis, normal or mildly increased protein concentration, and in 60% of patients, CSF-specific oligoclonal bands which were negative in our patient.^3^

 Until now, many cases of anti-NMDAR encephalitis have been reported worldwide and it is usually associated and triggered by ovarian teratoma in women and sometimes with testicular germ cell tumor in men.^3^ The second most common triggers of this autoimmune encephalitis are viral illnesses, which are more common in children.^8^ Although the herpes simplex virus is the most common trigger,^9^ other viral infections such as varicella zoster,^10^ measles,^11^ and parvovirus B19^12^ could also progress to anti-NMDAR encephalitis.

 There are many neurological and non-neurological complications that have been reported after COVID -19 vaccination such as headache, fever, myalgia, rash, weakness, and vomiting. Furthermore, there have been many more serious conditions such as Bell’s palsy, cerebrovascular accidents, multiple sclerosis relapse, fainting, as well as autoimmune and viral encephalitis following COVID-19 vaccination.^13-19^ Based on the following evidence, we have hypothesized that this diagnosed case of anti-NMDAR encephalitis might have been associated with the ChAdOx1 nCoV-19 vaccination:

 1) Some cases of anti-NMDAR encephalitis have been reported to arise from COVID-19 infection (notvaccination) in a person with no significant past medical history, and also in a psychiatric patient.^20-22^ Given the fact that ChAdOx1 nCoV-19 vaccine contains the SARS-CoV-2 structural surface glycoprotein antigen (spike protein; nCoV-19) gene, and consequently it can mimic the virus and possibly trigger the immune system in the same way, anti-NMDAR encephalitis following ChAdOx1 nCoV-19 vaccination seems to be a rational explanation.^23^

 2) Anti-NMDAR encephalitis has been also reported following Japanese encephalitis, tetanus, pertussis, polio, diphtheria, and H1N1 vaccinations. Interestingly, COVID-19 and these vaccines share some common biomarkers^24^; therefore, we could not rule out the possibility of anti-NMDAR encephalitis following COVID-19 vaccination.

 3) The patient did not have any other disease or medical disorders before receiving the vaccine, which could be another evidence in favor of this relationship.

 4) The described symptoms began only a few days after vaccination.

 5) The reported case had received a vaccine which stimulates the immune system, the use of immunosuppressive drugs attenuated his symptoms, and he has not had any other symptoms so far.

 6) Based on previous studies, anti-NMDAR encephalitis is usually associated with paraneoplastic situations,^3^ viral infections,^8^ or vaccines.^24^ The only stimulus that could be considered based on the history, physical examination, laboratory findings, and response to treatment in this patient was the vaccine that he was administered recently, increasing the risk of AstraZeneca vaccine to be associated with this anti-NMDAR encephalitis.

 In the end, it should be noticed that millions of people have used different vaccines without any complications, and these side effects are very rare. We reported a single case and propounded a hypothesis that the AstraZeneca vaccine could be the trigger of anti-NMDAR encephalitis in our patient, which could be helpful in rapid diagnosis and treatment of other possible future cases.
